# The Effect of Zbxz23ir-21 NANO(nanomaterials) Delivery Vector on Apoptosis and PTEN(phosphatase and tensin homolog deleted on chromosome ten)/PI3K(Intracellular phosphatidylinositol kinase)/AKT(related to the A and C kinase) in Children with CHOLESTEATOMA in Middle Ear

**DOI:** 10.1080/21655979.2021.1984718

**Published:** 2021-10-26

**Authors:** Hongwei Zheng, Wenlun Wang, Shichang Li, Lin Han

**Affiliations:** Department of Otolaryngology, Peking University People’s Hospital, Beijing, China

**Keywords:** NANO delivery vector, cholesteatoma in middle ear of children, apoptosis, PTEN/PI3K/AKT

## Abstract

Cholesteatoma of the middle ear is a kind of cystic disease with clear boundary formed by the abnormal growth of keratosquamous epithelium in temporal bone. Cholesteatoma otitis caused by it is a common disease in otorhinolaryngology. The EPR effect promotes the selective distribution of macromolecular substances in tumor tissues, which can increase drug efficacy. The purpose of this paper is to prepare and deliver the mir34a small molecule regulator, rubine, by nanotechnology, and to deliver it to the cells successfully. It can passively target tumor tissue through EPR effect, and play its regulatory role on miR-34a, thus inhibiting the growth of cholesteatoma cells. The effects of nano delivery on apoptosis and PIEN/P13K/AKt of children with middle ear choledochoma were tested in this paper. The experimental results were conducted on cholesteatoma cells as cell lines and balb/c nude mice as experimental objects. The expression of PTEN/PI3K/AKT in experimental group and control group was detected by immunohistochemistry. Apoptosis was discussed by cell activity detection. The physical and chemical properties, encapsulation efficiency, drug release ability in vitro and antitumor activity of nanoparticles in vitro and in vivo were studied. The results of cell level experiments in vitro showed that free RUBINE caused about 15% apoptosis, which was not different from RC NPs. The results showed that the nanoparticles could improve the expression of miR-34 in the cells, and then regulate the expression of Bcl-2, Cdk6 and CyclinD1, and play the inhibitory effect of miR-34a on the proliferation and migration of tumor cells.

## 1. Introduction

Cholesteatoma otitis media caused by middle-ear cholesteatoma is a common disease in otolaryngology [1,2]. Like tumors, cholesteatoma epithelial cells have high proliferative activity. Although there are many directions and controversies on the pathological mechanism of cholesteatoma, no theory can completely explain all the clinical features of cholesteatoma [3,4]. Because cholesteatoma also has many tumor-like characteristics, including abnormal proliferation, migration and invasion, the expression of miRNAs in cholesteatoma may change [5,6]. MiRNAs are involved in a series of life processes such as cell proliferation and apoptosis and has a very wide range of biological functions. In recent years, its research has become a hot spot, which also provides a new strategy for tumor treatment [7,8].

Many scholars have made unique achievements in the research of middle-ear cholesteatoma cells [9,10]. Some scholars have studied JAK/STAT signaling pathway in cholesteatoma. Eskiizmir and others found that STAT3 was positive in external auditory canal skin by immunohistochemistry but not in cholesteatoma tissue [11,12]. Some scholars also used Western blot to detect the expression of STAT3 in cholesteatoma and retroauricular skin and found that the expression of STAT3 in cholesteatoma was significantly higher than that in retroauricular skin, suggesting that the upregulation of STAT3 is related to the high proliferation of cholesteatoma epithelial cells [13,14].

The purpose of this article is to prepare and deliver the mir34a small molecule regulator, rubine, by using nanotechnology and to deliver it to the cells successfully. Apoptosis was discussed by cell activity detection. The physical and chemical properties, encapsulation efficiency, drug release ability in vitro and antitumor activity of nanoparticles in vitro and in vivo were studied. The main research work of this paper is as follows: first, define the related concepts of nanodelivery, middle-ear cholesteatoma cells, and PTEN/PI3K/Akt and discuss the related calculation formula of small molecule regulator rubone . Second, the effect of nanodelivery on apoptosis and Pien/P13 K/Akt of children’s middle-ear cholesteatoma cells was detected. BALB/C nude mice were selected as the experimental objects. Cell culture includes three processes: cell resuscitation, cell passage and cell cryopreservation. Third, the expression of PTEN/PI3K/Akt in the experimental group and the control group was detected by immunohistochemistry. The apoptosis was discussed by detecting the cell activity. The physicochemical properties, encapsulation efficiency, drug release ability in vitro and anti-tumor activity in vitro and in vivo of drug-loaded nanoparticles were studied.

## 2. Definition of relevant concepts

### 2.1 Cholesteatoma of middle ear

The clinical symptoms of cholesteatoma in the middle ear are pus in the ears, sometimes with erythema and blood, with specific odor. There is a marginal perforation at the relaxation of tympanic membrane or a marginal perforation on the top of the tense part. Sometimes, it can even be seen from the gap whether there is gray white bean rot residue sample in the drum room, and the odor is emitted [15].

### 2.2 The correlation between IL-21 (interleukin-21), STAT3 and P-STAT3 (phosphorylated-stat3) expression in cholesteatoma and granuloma and the relationship between IL-21, STAT3 and bone destruction

There was a significant positive correlation between IL-21 and STAT3 and p-STAT3 expression in cholesteatoma and granuloma tissues, suggesting that IL-21 may be acting on cholesteatoma and granuloma through STAT3 signaling pathway, but there was no significant difference in the positive expression rate of IL-21 in cholesteatoma and normal external ear canal skin tissue; therefore, it is not considered to have a significant effect on the proliferation of cholesteatoma keratinocytes, so IL-21 may play an important role in the chronic inflammation of the middle ear.

### 2.3 Nanocarriers

#### 2.3.1 Intracellular distribution of NANODRUG delivery system

With the development of the research, Nano Drug Delivery System (NDDSs) is influenced by cell types and its distribution and fate will be significantly different with the different endophagocytic pathways. Among them, the fate of NDDSs in the cell under the influence of different environment, the drug degradation, inactivation, activation and other different behaviors ultimately affect the effectiveness of the play. The different endogenosis pathway of NDDSs makes the distribution of NDDSs different and determines its fate in the cell. Therefore, it is very important to study and explain the distribution of NDDSs in cell, which is of great significance for the fate of drugs and the design of NDDSs.

#### 2.3.2 Advantages of NANO Carriers in drug delivery

The nano carrier has small size and good physical and chemical properties and has great advantages in the delivery of drugs: (A) It can improve the solubility of hydrophobic drugs in blood; (B) It can realize the delivery of drugs in cells; (C) Intelligent carriers, such as various stimulus and responsive intelligent carriers, can control drug release at a certain time in specific parts by using the specific stimulation and responsiveness of these vectors; (D) The method can be used for the joint transfer of various chemotherapeutic drugs, chemotherapeutic drugs and gene therapy drugs or disease treatment drugs and diagnostic measurement reagents and can avoid drug resistance of many drugs or realize the integration of diagnosis.

#### 2.3.3 Targeted modification of nanogene vector

Gene nanodrugs can play a role from entering the body to the focus, and it needs to go through five stages: (A) circulating in vivo with blood flow; (B) the tumor site was accumulated by EPR; (C) infiltrate into the solid tumor; (D) it enters the tumor cell through endocytosis; (E) the release of intracellular drugs was successfully carried out [16].

### 2.4 Rubone-related formula

#### 2.4.1 Rubone efficiency of RP NPS

The formulas of EE and LC are as follows:

(1) $$EE\left(\%\right)=\frac{W_{0}-W_{t}}{W_{s}}\times 100\%$$

(2) $$LC\left(\%\right)=\frac{W_{0}-W_{t}}{W_{0}}\times 100\%$$

where $W_{0}$ is the mass of initial rubone, $W_{t}$ is the mass of rubone in supernatant after centrifugation, $W_{s}$ is the mass of RP NPs after freeze-drying.

#### 2.4.2 In vitro release kinetics of RUBONE from RC NPS

The concentration of RUBONE was detected by HPLC, and the cumulative release rate of drug-loaded nanoparticles was calculated under different pH values. The formula was as follows (3), and the pharmacokinetic curve was drawn. The experiment was repeated three times and the average value of the measurement results was taken.

(3) $$E_{r}=\frac{V_{e}\sum_{1}^{n-1}C_{i}+V_{0}C_{n}}{m_{drug}}$$

where $E_{r}$ is the cumulative drug release, $V_{e}$ is the replacement volume of dialyzate, $V_{0}$ is the total volume of released medium, $C_{i}$ is the concentration of the released solution at the time of the i-th sampling, $m_{drug}$ is the total mass of drug in nanoparticles [17].

#### 2.4.3 Hemolysis rate of nanoparticles

Fresh blood was taken to remove serum. After washing with PBS for 3 times, the obtained red blood cells were dispersed into 10 ml PBS to obtain red blood cell dispersion. 0.5 ml red blood cell dispersions were added into 0.5 ml NANO particle solutions with different concentrations, and 0.5 ml red blood cell dispersions were added into 0.5 ml PBS and deionized water as negative control and positive control. The above mixture was vortex mixed, placed at room temperature for 3 h, centrifuged at 10,000 rpm for 5 min, and after taking a picture, take 100ml of each sample supernatant and add it to a 96-well plate, 3 multiple holes were set in each group, and the average value was taken. The formula of hemolysis rate is as follows:

(4) $$Hemolysis\left(\%\right)=\frac{A_{S}-A_{n}}{A_{p}-A_{n}}\times 100\%$$

Among them, $A_{S},A_{n},A_{p}$ was the absorbance of control group and experimental group [18].

#### 2.4.4 Cell mobility

The logarithmic MCF-7 cells were divided into two groups × 106/well was inoculated in the six well plate. When the confluence of cells reached 95%, the gun head was used to scratch and mark the position. Different transfection samples were added into the serum-free medium for transfection

(5) $$Migrationrate\left(\%\right)=\frac{Width_{1}-Width_{0}}{Width_{0}}\times 100\%$$

where $Width_{1}$ is the scratch width of cultured cells, $Width_{0}$ is the initial cell scratch width.

### 2.5 Cell survival rate

Generally, if three groups of experiments are set up, the cell survival rate formula is as follows..

(6) $$Viability\left(\%\right)=\frac{A-B}{C-B}\times 100\%$$

where A, B and C are the absorbance values of the control group and the experimental group, respectively.

### 2.6 Tumor volume

The two vertical diameters of the tumor were measured by vernier caliper, and the tumor volume was measured by the following formula.

(7) $$V=a\times b^{2}\times 1/2$$

where a is the length of the tumor and b is the short diameter of the tumor.

### 2.7 PTEN (Phosphatase and Tensin Homolog Deleted on Chromosome Ten)/PI3K (Intracellular Phosphatidylinositol Kinase)/AKT (Related to the A and C Kinase)

PI3K/Akt/mTOR signaling and transduction pathway is one of the oldest classical mediating pathways for cell survival. It is mainly responsible for participating in the internal and external signal transduction of human body and regulating the basic physiological processes of human cells, including cell cycle, proliferation, growth, apoptosis and cell survival. Phosphoinositide-3 kinase (PI3K/Akt/mTOR) is a kind of lipid kinase, which is an initiating factor of PI3K/Akt/mTOR pathway α, p110 β, p110 δ and P110 γ several subunits, which can be activated by cell surface receptors and are most widely studied in PI3K family. AK, also known as protein kinase B (PKB), is the core molecule of this pathway. It has a highly conserved pH (the pleckstrin homology) homologous structure region. After it is released into the cytoplasm, it phosphorylates the serine/threonine residues through specific action and mediates the interaction between protein and protein, thus participating in cell proliferation and apoptosis differentiation and apoptosis; p27,GSK-3 β, as a downstream target of Akt phosphorylation, can promote cell cycle progression.

### 2.8 Adsorption of bovine serum albumin by samples

The adsorption of RC NPs nanoparticles on the protein in plasma was measured by protein adsorption experiment using bovine serum albumin (BSA) as the model of plasma protein. The adsorption capacity of BSA can be obtained by the following formula.

(8) $$A=\left(C_{i}V_{i}-C_{s}V_{s}\right)/m$$

where a is the adsorption capacity of BSA on nanoparticles, $C_{i}$ is the initial concentration of BSA added to the sample, $C_{s}$ is the concentration of BSA in the supernatant after protein adsorption, $V_{s}$ is the total volume of solution after protein adsorption experiment, M is the mass of nanoparticles in solution.

### 2.9 Mir-34a inhibits tumor growth and metastasis in mice

After overexpression of miR-34a, cell proliferation was inhibited, and the expression level of EGFR was significantly decreased, which indicated that miR-34a inhibited the growth of mouse tumor. At the same time, E-cadherin increased and N-cadherin decreased, indicating that overexpression of miR-34a inhibits tumor metastasis.

### 2.10 Medical image enhancement theory

#### 2.10.1 Median filtering

Let f(x, y) denote the gray value of the image pixel, then the median filtering result is as follows:

(9) $$g\left(i,j\right)=Med\left\{f\left(i+s,j+t\right),\left(s,t\right)\in M\left(i,j\right)\right\}$$

#### 2.10.2 Unsharp mask

Let f(I, J) denote the gray scale of a pixel (I, J) in a certain gray image.

(10) $$g\left(i,j\right)=f\left(i.j\right)+k\left[f\left(i,j\right)-\overset{ˉ}{f}\left(i,j\right)\right]$$

where g(i, j) is the sharpened image and K is the magnification factor [19].

### 2.11 Blood compatibility of nanoparticles

Usually, after the carrier material enters the circulation of the body, nonspecific adsorption of protein will occur on the surface of the carrier material. The protein in the body will recognize and combine with the protein adsorbed on the surface of the carrier material and then cause inflammation, hemolysis, thrombosis or serious immune reaction, which makes the carrier function invalid. The less the amount of protein adsorbed on the surface of nanoparticles, the better the anti-protein adsorption performance. The hemolysis rate indicates the damage degree of erythrocyte membrane when the red blood cells contact with foreign bodies. The smaller the hemolysis rate is, the smaller the damage of nanoparticles to erythrocyte membrane is. When the hemolysis rate exceeds 5%, the material is considered to be hemolytic.

### 2.12 MiRNA targeted small molecule modulators

MiRNA regulation is equivalent to regulating multiple signaling pathways, which will involve and affect all stages of tumor development, such as tumor occurrence, development, metastasis and so on. Studies have confirmed that miRNA plays an important role in the occurrence and development of HCC. However, due to its easy degradation and low transfection efficiency, its practical application in tumor therapy is limited. Small molecular regulators that specifically target miRNA can avoid the problems of low transfection efficiency and easy degradation of miRNA. They can target miRNA in vivo and directly regulate the expression of miRNA, which provides a new way for tumor-targeted therapy [20,21].

## 3. Experiment

### 3.1 Experimental materials

#### 3.1.1 Main drugs and reagents

DMEM medium, phosphate buffer (PBS), HE kit, annexinv-pi apoptosis kit, DAPI, CCK-8 kit, GAPDH monoclonal antibody (mouse anti human), bcl-2 monoclonal antibody (mouse anti human), Cdk6 monoclonal antibody (mouse anti human), cyclinD1 monoclonal antibody (mouse anti human), real-time PCR primer, dimethyl sulfoxide (DMSO), BCA protein concentration determination kit, Ripa cell lysate were used.

#### 3.1.2 Cell lines

Cholesteatoma cells of middle ear in children were cultured in DMEM medium containing 10% FBS, 100 U/ml penicillin and 1 mg/ml streptomycin at 37°C, 5% CO_2_, saturated temperature and humidity; the cells could grow monolayer or multi-stage adherent.

#### 3.1.3 Laboratory animals

The healthy balb/c nude mice, about 4 weeks old, half male and female, weight 15–20 G. Nude mice were raised in an independent ventilation system (IVC) facility in SPF class laboratory. Group feeding, free drinking water, eating, temperature of 22.5 ± 2.5°C, humidity of 50–70%, air exchange velocity of 0.1–0.2 m/s, and the rest conditions are set conditions of ventilation system.

### 3.2 Experimental instruments

Tg-16 w high-speed centrifuge, kq-100 db numerical control ultrasonic cleaner, multifunctional microplate reader, laser confocal microscope, inverted fluorescence microscope, quantitative PCR instrument, vertical electrophoresis tank, flow cytometry were used.

### 3.3 Experimental methods

#### 3.3.1 Effect of NANO Delivery carrier on cell apoptosis

1) Cell subculture

Cell subculture refers to the static culture of cells at 37°C and 5% CO_2_. After the cells adhere to the wall, the culture medium is changed and the culture is continued.

2) Microscopic observation of the ability of labeled nanoparticles to enter children’s middle ear cholesteatoma cells.

Because the nanomaterials used in this experiment have no fluorescence characteristics, we use CY-3 to label the nanomaterials. The method is as follows: 1 mg RP NPs nanoparticles were dissolved in 5 ml ultra pure water, and 200 mg RP NPs nanoparticles were added. 1 mg/ml of lcy-3-nhs in DMSO solution was stirred at room temperature for 12 h in an inert atmosphere and dark conditions, centrifuged, washed with ultrapure water for three times, and then the surface of CMD was modified with cy-3-labeled RP NPs.

The labeled RC NPs nanoparticles were added into children’s middle ear cholesteatoma cells, and the nucleus and lysosome were stained with DAPI staining solution and lysotracker green staining solution, respectively. The distribution of RC NPs in children’s middle ear cholesteatoma cells was observed by laser confocal microscopy. The specific operation steps are as follows: 1 × Methods: 105 middle ear cholesteatoma cells were seeded in a confocal dish and cultured in DMEM medium 37°C. The cells were cultured in 5% CO_2_ and saturated humidity incubator overnight; the cultured cells were washed with PBS for 3 times, and then the drug-loaded nanoparticles RC NPs labeled with CY-3 were added 37° C. .Choosing 5% CO2 and place it in a saturated humidity incubator for 6 hours, suck up the medium and wash the PBS 3 times; fix each plate with 1 ml 4% formaldehyde fixative for 20 min, then remove the fixative and wash  with PBS for 3 times. Each dish was added with 1 ml of DAPI staining solution for 10 min, then DAPI staining solution was removed and washed with PBS for 3 times for 2 min each time. Then, each dish was added with 1 ml lysotracker green staining solution for 10 min, lysotracker green staining solution was removed and washed with PBS for 3 times for 2 min each time; the distribution of labeled nanoparticles in cholesteatoma cells was observed by microscope.

3) The cytotoxic effect of different component nanoparticles by CCK-8 experiment

CCK-8 reagent was used to determine the toxicity of the nanoparticles of the original drug, blank nanoparticles and drug carriers to the cholesteatoma cells in children.

4) Western blot detection of Bcl-2, cyclin D1 and Cdk6 protein expression

The expression of miR-34a target gene was detected by Western blot. The experimental group was divided into free rubine, BC NPs, RC NPs and negative control group (PBS), and the amount of addition of the experimental group was 20 according to the rubine concentration µ M calculation.

5) Cell scribing experiment

The effect of drug-loaded nanoparticles on cell migration was measured by cell line test. The specific experimental steps are as follows: (A) inoculate HepG-2 cells on the 6-hole plate, 6 for each hole × 105 cells, at 37°C. The cells were attached to the wall by culture for 24 hours in a 5% CO2 and saturated humidity incubator; (B) When the cell convergence degree reaches about 80%, the culture medium is drawn out, and a scratch is made on the cell with tip head in the center of the hole. The cells are observed and photographed under the inverted microscope (0 hour at time point), and the mark on the edge of the scratch is taken as the data test point; (C) Free rubine, BC NPs and RC NPs were added, respectively, and the concentration of rubine was 20 µ M. At the same time, the blank control group was set up with 3 multiple holes, and after 24 hours culture, the cells were observed and photographed under the inverted microscope (24 hours at the time point), and the distance between the cells on both sides of the scratch was measured.

6) Detection of miR-34a and its target gene expression by QRT PCR

The effect of different components on miR-34a and its target gene expression in children with middle ear choledochoma cells was detected by QRT PCR. The experimental group was divided into free rubine, BC NPs, RC NPs and negative control group, and the amount of addition of the experimental group was 20 according to the rubine concentration µ M calculation.

7) Western blot detection of Bcl-2, cyclin D1 and Cdk6 protein expression

The expression of miR-34a target gene was detected by Western blot. The experimental group was divided into free rubine, BC NPs, RC NPs and negative control group (PBS), and the amount of addition of the experimental group was 20 according to the rubine concentration µ M calculation.

a. HepG-2 cells were collected in logarithmic growth stage and inoculated on 6-orifice plate, and the number of cells per hole was 5 × 105 cells, and the cells were cultured overnight to make the cells adhere to the wall.

b. The free rubone and RC NPs were mixed with DMEM medium of 10% (v/v) FBS, respectively. The 6-hole plates were added; each group was set with 3 multiple holes, 37°C cultured cells for 48 hours.

c.Use trypsin digestion to separate and collect the cells, place them in a 1.5 ml centrifuge tube, add 100 μL of ripa cell lysate (containing 1 mm PMSF), use a pipette to blow away the cells to make them evenly distributed, after 5 minutes of division,  place it on ice.

d.Centrifuge the lysed cell suspension in an incubator at 14,000 rpm and 4°C for 10 minutes.

e. The total protein concentration was determined by BCA method;

f. The expression level of target protein was detected by Western blot: SDS PAGE, transmembrane and development.

8) Tumor suppression experiment in vivo

a. The model of middle ear bile tumor in children was established: 20 balb/c nude mice weighing 20 g were selected as experimental mice. 100μL of cells in the right back of each nude mouse contains 5×106 HepG-2 cells in normal saline. When the tumor size is 100 mm3, treatment will be started about 2 weeks later.

b. The nude mice were divided into four groups, each group was divided into 5 mice. Saline, free rubine, BC NPs and RC NPs were injected into nude mice inoculated with tumor via tail vein, respectively. The amount of rubone is 10 mg/kg. Every 3 days, the tail vein was administered once, 5 times in total.

c. The two diameters of the tumor vertical direction of nude mice were measured by vernier calipers, and the tumor volume was calculated according to formula (2).

d. After 5 times of administration, the nude mice were killed, the tumor tissue was taken out, weighed and photographed. The tumor weight was used as the histogram of tumor quality change in each experimental group. The tumor tissue was divided into two parts, one was used to detect miR-34a and its target gene expression level in tumor tissue, and the other was used for HE staining analysis of tumor tissue sections. Meanwhile, the heart, liver, spleen, lung and kidney were taken out for slice staining analysis.

9) In vivo imaging experiment

The distribution of Cy3 labeled RC NPs in nude mice was observed by fluorescence imaging. The experimental steps were as follows: (a) the tumor model was established step by step, and the mice were cultured until the tumor size was about 300 mm^3^; (b) Cy3-labeled RC NPs 100 was injected into mice via tail vein μ g; (c) the distribution of Cy3 labeled RC NPs in mice was observed. The injection time was 0 h, and the observation time points were 1 h, 6 h, 12 h and 24 h after injection; (d) After 24 hours of injection, the mice were euthanized, and the tumor, heart, liver, kidney, spleen and lung of the mice were taken out. The distribution of Cy3 labeled nanoparticles in various organs of the mice was observed by rat/mouse bright field and fluorescence imaging system.

#### 3.3.2 Effect of nano delivery carrier on PTEN/PI3K/Akt

1) Experimental materials

Polyethyleneimine polyester copolymer, (pei-pcl: Pei 800 Da, PCL 20 kDa), carboxymethyl dextran (CMD, 27 kDa), 2‘-hydroxy-2,4,4ʹ,5,6‘-pentamethoxychalcone, (rubone, 374 DA), polyvinyl alcohol (PVA, 89 ~ 98 kDa), block polyether (F-68, 8300 DA), chloroform, dimethyl sulfoxide, uranyl acetate, acetonitrile were used.

2) Experimental steps

A. Preparation of pathological sections: there were 71 cases in the experimental group and the control group. They were divided into p-Akt group, p-mTOR group, p-4ebp1 group and PTEN group. Before the immunohistochemical experiment, they were heated in 37°C oven overnight.

B. The cells were analyzed immunohistochemically.

C.Cell treatment process: first culturing the cells, and then observing and processing the cells. Then the cells were treated. The adherent cells were gently washed once and twice with PBS buffer; after adding appropriate amount of trypsin and observing carefully under the microscope, the shape of cells in the body was changed to ellipse; gently blow the cells with a dropper to make all the cells fall out or fall off from the culture bottle wall as far as possible, transfer the cells to the open culture bottle, centrifuge for 2 min at 1000 RMP; discard the upper layer cell culture medium, add the lower layer cell frozen storage medium, gently resuspend, and continue to blow even cells, using the lower layer cell counting plate, adjust its concentration to 3×. After 104 cells/ml, NSCLC cells were seeded on 96 well plates with 100 cells per well μ 50. After 24 hours of culture, the cells were treated with different concentrations of DMSO solution or ethanol solution, and the blank group was set up with 100 cells per well μ 50. After 24 hours of culture, different concentrations of ethanol and zedoary turmeric alcohol solution prepared with different concentrations of ethanol were added. At the same time, blank group was set up (no cells were inoculated, no zedoary turmeric alcohol or solvent was added, and other parallel operations were performed). The system of each well was 100 μ 50. MTT cytotoxicity test was carried out at 37°C and 5% CO2 for 24 h.

The last is MTT cytotoxicity test.

3) Experimental data collection

First, place each slice in a 400×500 optical microscope field of view, and randomly select 200 sample slices for photographing. Then the patients were asked by telephone or consulted the inpatient information to understand the age, gender, disease staging, lactate dehydrogenase level at the initial diagnosis, tumor location, physical status, number of extranodal invasion, IPI score, time of death or loss of follow-up.

4) Immunohistochemical control

Positive control: four sections of extranodal nasal type NK/T lymphoma with known PTEN antibody positive, p-Akt antibody positive, p-4ebp1 antibody positive and p-mTOR antibody positive were used as positive control. The results were positive.

Negative control: four sections of extranodal nasal type NK/T lymphoma with known PTEN antibody negative, p-Akt antibody negative, p-4ebp1 antibody negative and p-mTOR antibody negative were used as negative control. The results were negative.

Blank control: four sections of extranodal NK/T lymphoma with known PTEN antibody positive, p-Akt antibody positive, p-4ebp1 antibody positive and p-mTOR antibody positive were incubated with PBS instead of primary antibody as blank control. The results were negative.

## 4. Results and discussion

### 4.1 Effect of NANO Delivery carrier on apoptosis

#### 4.1.1 Cellular uptake and intracellular distribution of RC NPS

In the previous section, RC NPs were labeled with CY-3 to make them have red fluorescence, and the nucleus and lysosome were stained with DAPI staining solution (blue fluorescence) and staining solution (green fluorescence) respectively. The uptake and distribution of RC NPs in cells were observed by laser confocal microscope. The results showed that a large number of red fluorescence (cy-3-rc-nps) appeared in the cytoplasm of cells and separated from green fluorescence. These results indicate that RC NPs can be effectively absorbed by cells and escape from lysosomes into cytoplasm after entering cells.

The purpose of this paper is to prepare and deliver the mir34a small molecule regulator, rubine, by nanotechnology and to deliver it to the cells successfully. Apoptosis was discussed by cell activity detection. The physical and chemical properties, encapsulation efficiency, drug release ability in vitro and antitumor activity of nanoparticles in vitro and in vivo were studied.The results showed that the nanoparticles could improve the expression of miR-34 in the cells, and then regulate the expression of Bcl-2, Cdk6 and CyclinD1, and play the inhibitory effect of miR-34a on the proliferation and migration of tumor cells.

#### 4.1.2 Effects of drug-loaded nanoparticles on cytotoxicity, apoptosis and migration

**Figure 1.**
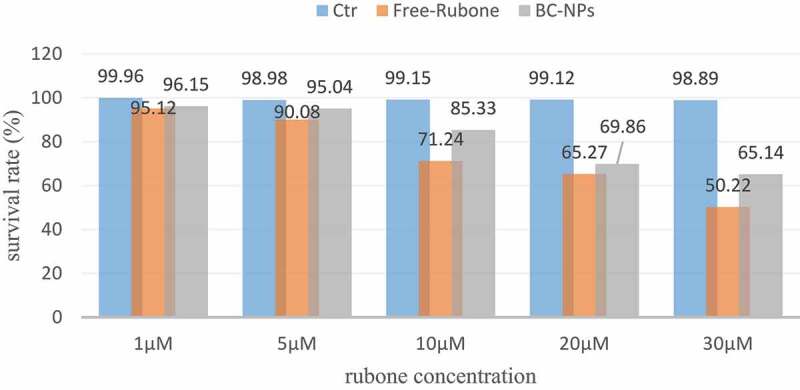
Cell viability at different RUBONE concentrations

The results of laser confocal experiments show that nanoparticles can enter the cells. We further determine the effects of the original drug, blank nanoparticles (BC NPs) and drug-loaded nanoparticles (RC NPs) on the survival rate of the children with middle ear cholesteatoma cells. As shown in Figure 1, we set 1, 5, 10, 20, 30 at rubine concentration in the experiment µ. The cell survival rate of different rubone concentrations was measured by CCK-8 method at 24 hours.

The experimental results shown in Figure 1 show that (a) free rubone and RC NPs can significantly inhibit the proliferation of cholesteatoma cells in children’s middle ear; (b) The cell survival rate decreases as the concentration of RUBONE increases. It can be seen that Rubone has a strong killing effect on cells. When the concentration of Rubone is 30 μM, the cell survival rate is about 60%, which is not much different from when the concentration of Rubone is 20 μM; (c) The inhibition effect of free rubone on the proliferation of cholesteatoma cells in children is slightly better than that of RC NPs, which is due to the slow release of drugs in nanoparticles.

**Figure 2.**
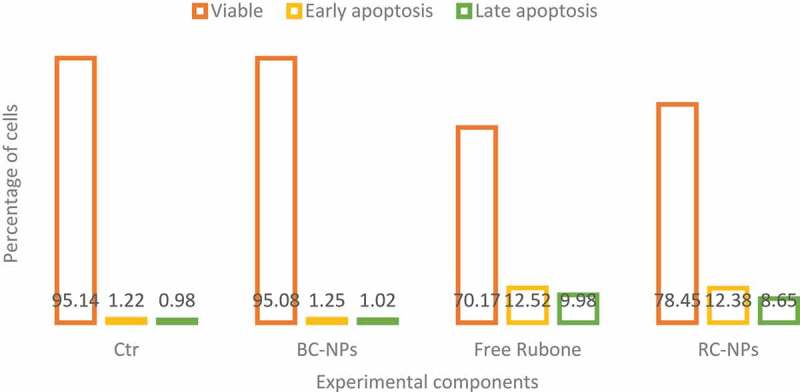
Apoptosis of cells treated with different components

We treated children’s middle ear cholesteatoma cells with different components. The dose of rubone in the experimental group was 20 µ M. Apoptosis was detected by flow cytometry. The results are shown in Figure 2.

As shown in Figure 2, similar to the results of cytotoxicity test, the control group and BC NPs did not cause obvious apoptosis of cholesteatoma cells in children’s middle ear. In the experimental group, both free rubone and RC NPs could induce apoptosis. As can be seen from the figure, free rubone caused about 15% of the apoptosis, including early and late apoptosis, which was similar to that caused by RC NPs.

Finally, we detected the migration ability of HepG-2 cells treated with different experimental components by cell scratch test. Taking PBS as negative control group, BC NPs, free rubone and RC NPs were co-incubated with HepG-2 cells. The migration ability of HepG-2 cells treated with free rubone and RC NPs was observed under inverted microscope at 0 h and 24 h, respectively; these results indicate that free rubone and RC NPs can inhibit the migration of cholesteatoma cells in children.

#### 4.1.3 Regulation of miRNA-34a and its target gene expression by drug-loaded nanoparticles

**Figure 3.**
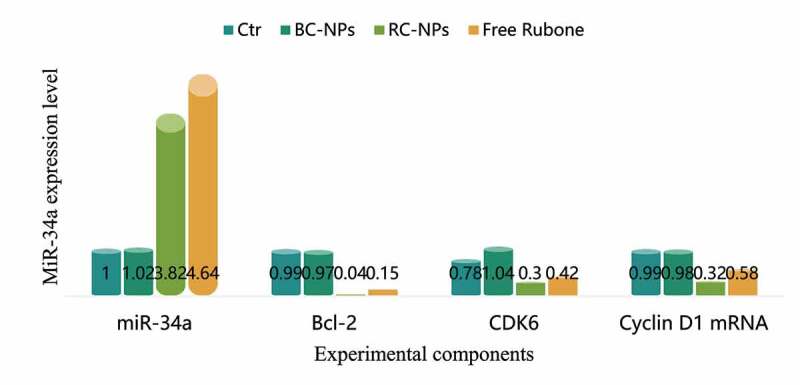
MiR-34a expression level

MiR-34a is a broad-spectrum tumor suppressor gene. It can regulate many target genes, including Bcl-2, Cdk6, CyclinD1, etc. by regulating these target genes, it can induce apoptosis, inhibit cell proliferation and migration. However, the low expression of miR-34a in most tumors, including hepatocellular carcinoma, leads to the high expression of its target gene, which inhibits the apoptosis of cancer cells and enhances the proliferation and migration ability. Therefore, upregulation of miR-34a level to restore its tumor inhibitory function has become a new strategy in tumor therapy. In this study, the expression of miR-34a can be regulated by delivering small molecule miR-34a regulator rubone into cells, which can induce apoptosis and inhibit cell proliferation and migration. The expression of miR-34a, Bcl-2, Cdk6 and cyclin D1 mRNA in children’s middle ear cholesteatoma cells transfected with rubone nanoparticles is shown in Figure 3.

As shown in Figure 3, the expression level of miR-34a in the control group and BC NPs group was basically the same, and there was no significant change. The expression level of miR-34a in free rubone group and RC NPs group was significantly increased, indicating that drug-loaded nanoparticles can deliver rubone into cells and up-regulate the level of miR-34a in cells. The expression level of miR-34a in free rubone group was slightly higher than that in RC NPs group, which was due to the slow and controlled release of nanoparticles. Compared with the control group and BC NPs group, the expression levels of Bcl-2, Cdk6 and cyclin D1 mRNA in free rubone group and RC NPs group were significantly lower.

We further detected the mRNA expression levels of miR-34a target genes Bcl-2, Cdk6 and CyclinD1. As shown in Table 1.

**Table 1.** Expression levels of Bcl-2, Cdk6 and CyclinD1 mRNA

	Bcl-2	CDK6	CyclinD1mRNA
CTR	0.98	0.98	0.98
BC-NPs	0.96	0.99	0.97
Free RUBONE	0.095	0.29	0.31
RC-NPs	0.12	0.46	0.58

Compared with the control group and BC NPs group, the expression levels of Bcl-2, Cdk6 and cyclin d1 mRNA in free rubine group and RC NPs group were significantly lower.

#### 4.1.4 In vivo antitumor activity of drug loaded nanoparticles

**Figure 4.**
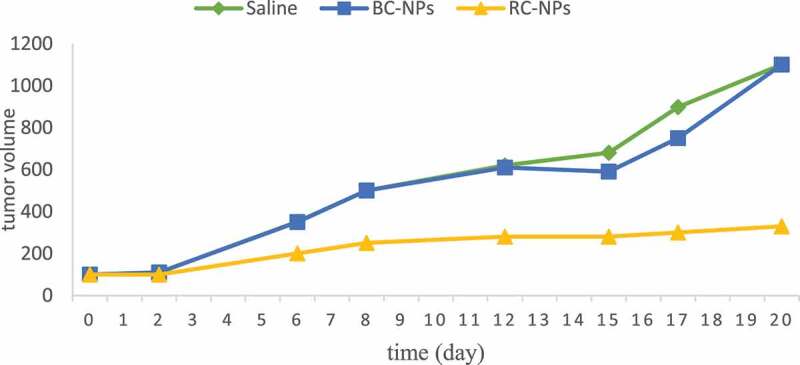
The volumes of tumors

The above studies have proved that RC NPs can enter into cells in vitro and significantly inhibit the growth of HepG-2 cells. Furthermore, we established a tumor model of liver cancer and studied the inhibitory effect of different components of nanoparticles on tumor. After HepG-2 cells were inoculated, the nude mice were divided into 4 groups with 5 mice in each group when the tumor volume reached about 100 m3. Saline, BC NPs and RC NPs were injected into nude mice via tail vein. The dosage of rubone was 10 mg/kg. The injection was carried out every three days, five times. Before each administration, the tumor volume was measured with vernier caliper, and the tumor growth curve was drawn. After the treatment, the nude mice were killed, the corresponding tumor tissues were taken out, photographed and weighed. Figure 4 shows the growth curve of the tumor during administration and the picture of the tumor after treatment.

It can be seen from the figure that the tumor volume of saline control group and BC NPs group increased significantly with time, indicating that BC NPs had no anti-tumor activity. Compared with saline control group and BC NPs group, the increase of tumor volume of RC NPs group was significantly slower, indicating that it could inhibit the growth of tumor.

#### 4.1.5 Expression levels of miR-34a and its target genes in tumor tissues

**Figure 5.**
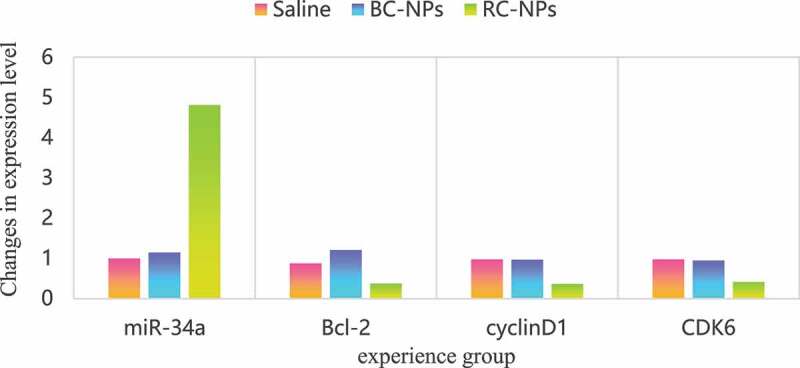
Expression levels of miR-34a, Bcl-2, CyclinD1 and Cdk6 in tumor tissues

The changes of miR-34a and its target gene expression levels in tumor tissues were further studied by QRT PCR experiment. The changes are shown in Figure 5.

It can be seen from Figure 5 that compared with saline control group and BC NPs group, miR-34a expression level in RC NPs group was significantly upregulated, while the expression of Bcl-2, cyclin D1 and Cdk6 was significantly inhibited, and the expression level was downregulated. This indicated that rubone could be successfully delivered to the tumor site by tail vein injection, it can play a role in regulating miR-34a in tumor cells, improve the expression of miR-34a in tumor site, restore the inhibitory function of miR-34a on tumor, and then inhibit the growth of tumor.

#### 4.1.6 Toxicity of nanoparticles to main organs and tissues of nude mice

In order to study the effect of drug loaded nanoparticles on main organs and tissues, after treatment, the main organs of nude mice in each group were taken out for paraffin section and he staining to observe whether the tissue morphology changed. Compared with saline control group, the histological structure of heart, liver, spleen, lung and kidney in BC NPs group and RC NPs group were normal. These results indicate that the delivery of rubone into the body by RC NPs can achieve better tumor treatment effect without obvious systemic toxicity.

### 4.2 Effect of NANO delivery carrier on PTEN/PI3K/AKT

#### 4.2.1 Expression of P-AKT in children’s middle ear CHOLESTEATOMA cells

Immunohistochemical results showed that the expression rate of p-AKT in CHOLESTEATOMA cells was 82.69% (43/52), and the expression rate of p-AKT in reactive proliferative lymph nodes was 22.12%. There was significant difference between the two groups (x2 = 21.11, P < 0.005), as shown in Table 2.

**Table 2.** Expression of p-AKT in CHOLESTEATOMA cells of middle ear in children

Group	N	p-AKT					Positive rate	
		-	+	++	+++	%/	$x^{2}$	p
Experience group	52	9	18	16	9	82.69	22.11	
Control group	20	15	4	1	0	22.12		

#### 4.2.2 Expression of PTEN gene in CHOLESTEATOMA cells of middle ear in children

The results of immunohistochemistry are shown in Table 3, and the difference was statistically significant (x2 = 12.06, P < 0.005). The expression of PTEN gene was mainly concentrated in cytoplasm.

**Table 3.** Expression of PTEN in middle ear CHOLESTEATOMA

Group	N	PTEN					Positive rate	
		-	+	++	+++	%	$x^{2}$	p
Experience group	52	32	11	7	2	39.21	12.06	
Control group	20	3	7	6	4	85.12		

## 5. Conclusion

This paper mainly discusses the effect of nano delivery on apoptosis and PTEN/P13 K/Akt of cholesteatoma cells. In the aspect of cell apoptosis, it has been found that nano delivery has a great inhibitory effect on cell proliferation. As for signal transduction pathway, its main role in cells is to regulate and promote the translation of cyclin, which plays an important role in cell proliferation, growth, apoptosis, angiogenesis and so on. This study shows that there are also signaling pathways in cholesteatoma cells, such as DLBCL, MCL, ALCL and HL. Experiments show that miR-34a can inhibit the proliferation, migration and cell cycle of NSCLC cells and promote apoptosis. In vivo experiments show that miR-34a can inhibit tumor growth and metastasis. This indicates that miR-34a may act as a tumor suppressor by targeting EGFR.

## Figures and Tables

**Table:** 

**Abbreviation**	**Full name**
Bcl-2	B-cell lymphoma-2
EGFR	epidermal growth factor receptor
NSCLC	nonsmall-cell lung cancer
